# Obesogenic Environments: Access to and Advertising of Sugar-Sweetened Beverages in Soweto, South Africa, 2013

**DOI:** 10.5888/pcd12.140559

**Published:** 2015-10-29

**Authors:** Gillian Moodley, Nicola Christofides, Shane A. Norris, Thomas Achia, Karen J. Hofman

**Affiliations:** Author Affiliations: Gillian Moodley, School of Public Health, Faculty Health Sciences University of Witwatersrand, Priority Cost Effective Lessons for Systems Strengthening, Johannesburg, South Africa; Nicola Christofides, Thomas Achia, School of Public Health, Faculty Health Sciences University of Witwatersrand, Johannesburg, South Africa; Shane A. Norris, Faculty Health Sciences University of Witwatersrand, MRC/Wits Developmental Pathways for Health Research Unit, Department of Paediatrics, School of Clinical Medicine, Johannesburg, South Africa.

## Abstract

**Introduction:**

Rates of obesity and overweight among South Africans are increasing. Food marketing has a profound impact on children and affects their lifelong eating patterns; in urban areas of South Africa, disposable incomes are growing and ultra-processed food is increasingly available at low cost. The combination of these factors will strain an already fragile health system. Our aim was to investigate the density of outdoor sugar sweetened beverage (SSB) advertising and the number of formal and informal vendors selling SSBs in a transforming, historically disadvantaged urban setting of South Africa.

**Methods:**

A digital camera and global positioning system navigation system were used to record the location of SSB advertisements and food vendors in a demarcated area in Soweto. Data were collected by walking or driving through each street; a food inventory was completed for every food vendor. Spatial analyses were conducted using a geographic information system.

**Results:**

A total of 145 advertisements for SSBs were found over a driven or walked distance of 111.9 km. The density of advertisements was 3.6 per km^2^ in relation to schools, and 50% of schools had branded advertising of SSBs on their school property. Most (n = 104; 58%) of the 180 vendors in the study sold SSBs.

**Conclusion:**

This is the first study in South Africa to document the location of billboard advertisements and vendors in relation to schools. Marketing of products that contribute to obesity is common in urban Soweto. Our findings have implications for policies that regulate SSB advertising, especially in the proximity of schools.

## Introduction

Non-communicable diseases (NCDs) will be the leading cause of death on the African continent by 2030 (1). Between 1992 and 2005, obesity prevalence increased by 35% in sub-Saharan Africa (2). In 2012, South Africa had an obesity prevalence of 39.2% among females and 10.6% among males (3). The level of obesity among adolescent girls has increased significantly; 25% of female adolescents are overweight or obese (4,5).

Most South Africans have poor dietary habits, many of which start during childhood; black South Africans have the lowest dietary diversity of all South Africans and a higher-than-average sugar intake (3). Analysis of sugar sweetened beverage (SSB) consumption in Soweto indicated that adolescents consume between 1.1 and 1.4 servings of SSBs daily (6). This amount translates into 10 to 12 teaspoons per day, which exceeds the proposed World Health Organization daily recommendation of 6 teaspoons of sugar per day (7). This high sugar intake from a single source significantly increases the risk of developing obesity-related NCDs, especially type 2 diabetes (6). Furthermore, sugar has been implicated as a contributor to obesity (8).

A combination of rising incomes and discretionary spending, coupled with marketing, advertising, and availability of high-energy, processed food and beverages, the biggest source of added sugar, play a role in fostering this trend (3). The increasing consumption of processed products is linked to commercial advertising and their greater availability. Marketing by the food and beverage industry strongly influences long-term food and beverage preferences, and its success relies on children’s brand recognition and subsequent preference for familiar brand foods (9–11).

Neighborhoods of various socioeconomic statuses have different levels of exposure and intensity to advertisements of ultra-processed food and beverage products (10,11). The effect of advertising on creating and promoting an obesogenic environment has been demonstrated in the United States, where a 30% increase in food advertising resulted in increased obesity levels, and every 10% increase in the number of fast food advertisements was accompanied by a 6% increase in the consumption of SSBs (11). The higher density of advertisements of unhealthy foods in low-income areas was accompanied by an absence of exposure to goods and activities that promote healthier lifestyles (11,12).

In addition to selling SSBs, street vendors in South Africa sell other high-sugar–content items, such as candy and deep-fried doughnuts known as “vetkoek” (13). Other easily available street foods include burgers, deep-fried potato chips, and “kotas” (a quarter loaf of bread with a combination of deep fried chips, cheese, and meat fillings) (13). Advertising and access to obesity-promoting beverages and street foods contribute to obesity in South Africa (10).

Limited data are available on the density of outdoor advertising and vendors in South Africa. This study examined 2 aspects of the obesogenic environment in an urban setting in South Africa by exploring the frequency and location of outdoor advertising for SSBs and the proportion of food vendors selling SSBs. The goal was to understand how to best craft advocacy activities that limit the promotion of unhealthy products, particularly in settings in close proximity to schools.

## Methods

Soweto is a historically disadvantaged area of Johannesburg that covers more than 200 square kilometers and has a population of 1.3 million. There are 1,776 households and 6,357 inhabitants per square kilometer (14). During the past decade, Soweto transformed economically; by 2013, four large shopping malls and several fast-food chains entered the market. This study covered 5 areas in Soweto: Klipspruit West, Mofolo South, Dube, Meadowlands, and Orlando East. During July and August 2013, data on all outdoor SSB advertising and SSB branding in a 38.3 km^2^ area were collected.

Ethics approval was granted by the University of the Witwatersrand Ethics Committee. The study did not include human participants, and both the advertisements of SSBs and the food and beverages being sold by vendors were in the public domain.

Data were collected by 3 trained research assistants who either walked or drove through each street in the study area. SSB advertisements and food vendors were identified, and data were collected using 2 separate data coding sheets. In addition to collecting information on the location (global positioning systems [GPS] coordinates were determined using a Garmin Nuvi 30, Garmin Ltd.), type, and size of the advertisement, a digital photograph was taken using a Sony Cybershot DSC-W270 12.1 megapixel camera (Sony Corporation). The study team completed an inventory of all food and drink items for sale from vendors. The type of vendor was noted as informal (ie, temporary building structure), formal (ie, permanent building structure with limited resources, for example, no access to electricity or refrigeration), or a shop (ie, permanent structure with access to electricity and refrigeration), and an inventory of beverages sold at these vendors was recorded.

For the purpose of this study, outdoor advertising was defined as billboards, bus stop advertisements, signs placed along the sidewalk, urban art on streets or buildings, large posters, and signage for restaurants or food vendors. Items excluded were branded clothing, packaging, and taxis and buses (ie, moving targets). Advertisements of SSBs and a combination of SSBs and fast foods were included in the analysis.

Researchers were unable to measure the size of the advertisements, so an estimation of each advertisement size was made. Advertisements were classified as small if their dimensions were less than approximately 70 by 40 centimeters or, in terms of paper and cardboard sizes, between A4 (8.3 × 11.7 in) and A3 (11.7 × 16.5 in). Medium advertisements had dimensions of A0 (33.1 ×46.6 in) and large advertisements had dimensions that were measured in meters (eg, billboards).

The GPS coordinates of SSB advertisements and vendors in the study area were used to create distinct spatial point pattern objects in the R library Spatstat (www.spatstat.org). A point pattern that consisted of all the advertisements and vendors in the study area was also created. Using Spatstat, the intensity (number of points per km) of each point pattern was computed. The intensity of each point pattern formed the outcome variables in the spatial point pattern analysis. To assess the association between the point patterns and the distribution of schools in the study, a covariate that measured distance from any given point in the study region to the nearest school was created using the distmap function in Spatstat. We assessed the association between the intensity of each point pattern and the covariates of interest using the Kolmogorov–Smirnov test of goodness-of-fit (15,16).

Homogeneous and inhomogeneous Poisson models were fitted using the R library Spatstat (15–19), and the Akaike Information Criterion was used as a basis for selecting the best fitting model (20,21). In each model, the distance to the nearest school (*z*) was the primary covariate considered. Models 1, 2, and 3 were best-fitting inhomogeneous Poisson models that were fitted to the SSB, vendor, and both SSB and vendor point patterns, respectively. A fourth model (Model 4) was an inhomogeneous Poisson model with marks labeled as SSB and vendors.

## Results

In total, 145 advertisements for SSBs were identified (Table 1). More than half (53%) of SSB advertisements were found outside houses. Many informal vendors operated stalls from their homes. Nearly two-thirds (62%) of branded SSB advertisements were part of a display sign for a shop name, including branded signs for tuckshops (ie, small shops located in or near a school that sell snacks, candies, beverages, and food items that target children) found outside houses. Half of the primary and high schools (14 of 28) in the sample area displayed advertisements of SSBs on school premises, and 13 of these were branded school signs.

**Table 1:** Size, Location, and Format of Sugar-Sweetened Beverage (SSB) Advertisements (N = 145), Soweto, South Africa, 2013

Characteristic of advertisement	n (%)
**Location**	
House	77 (53.1)
Small shopping centre	23 (15.9)
Street/side of road	20 (13.8)
Primary or high school	13 (9.0)
Other building	7 (4.8)
Shebeen	3 (2.1)
Crèche/preschool	1 (0.7)
Transport hub/taxi stand	1 (0.7)
**Format**	
Shop sign	79 (54.5)
Poster	24 (16.6)
Painted advertisement	15 (10.3)
School sign	12 (8.3)
Bus stop	6 (4.1)
Banner	3 (2.1)
Pole sign	3 (2.1)
Billboard	2 (1.4)
Umbrella/refrigerator	1 (0.7)
**Size**	
Small	11 (7.6)
Medium	94 (64.8)
Large	37 (25.5)
Large billboard	3 (2.1)

A total of 180 vendors were included in the study area; 27% were informal fast-food outlets, 12% were formal outlets, and 61% were shops. More than 85% of shops stocked SSBs (Table 2). Formal and informal vendors both supplied fast food, although few of these vendors stocked SSBs because of lack of refrigeration.

**Table 2:** Vendors (N = 180) That Sold Sugar-Sweetened Beverages (SSBs), Diet SSBs, Fruit Juice, and Milk, by Vendor Type, Soweto, South Africa, 2013

Vendor Type	No. of Vendors	No. of Vendors That Sold Beverage Type			
		SSBs	Diet SSBs	Fruit Juice	Milk
Informal (housed in a nonpermanent structure)	49	1	0	0	0
Formal (housed in permanent structure with no access to electricity/refrigeration [eg, spaza shop])	22	3	0	0	0
Shops (housed in a permanent structure with access to electricity/refrigeration)	109	100	40	48	74
Percentage of vendors that sold the item	NA	58	22	27	41

**Figure 1:**
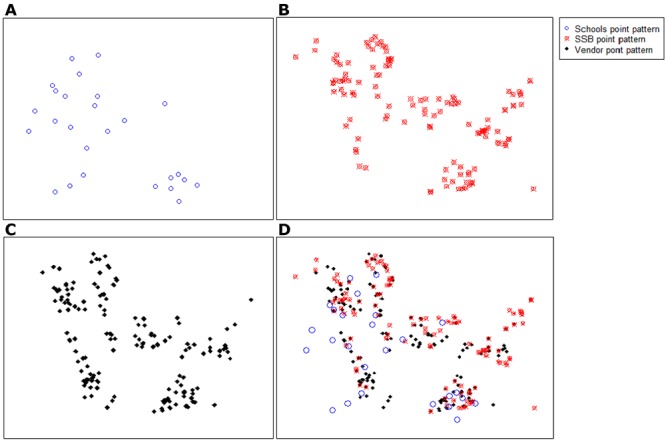
Kernel density and contour plots, demonstrating the density of sugar-sweetened beverage (SSB) advertisements and their distances to schools and vendors, Soweto, South Africa, 2013. Graph A shows the school point pattern, Graph B shows the SSB point pattern, Graph C shows the vendor point pattern, and Graph D shows all point patterns.

**Figure 2:**
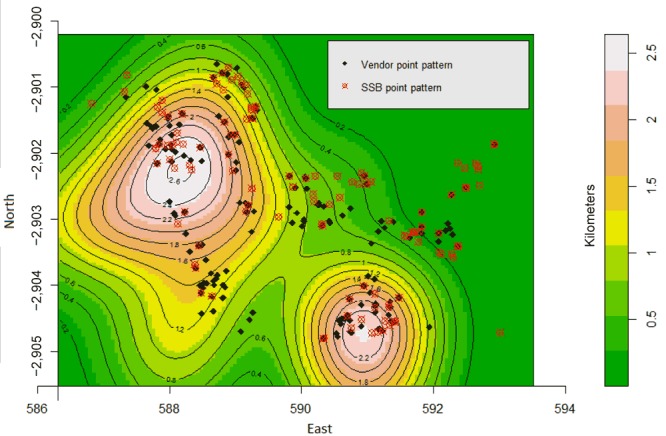
Kernel density and contour plots of school point pattern overlayed on vendor and sugar-sweetened beverage point patterns, Soweto, South Africa, 2013.

The findings from the spatial analysis described the intensity of SSB advertisements and vendors in relation to schools. The intensity of the SSB point patterns in the study area of 38.3 km^2^ was 3.58 points per square kilometer (Table 3). Figure 1 depicts the density of SSB advertisements and their distances to schools and vendors. The figures indicate 2 school clusters in the northwestern and southeastern parts of the study area. The vendor and SSB advertisements identified were distributed around school “hotspots” (Figure 2).

**Table 3:** Intensity of Advertisements (Points/km^2^) Versus No. of Schools, Sugar-Sweetened Beverage (SSB) Advertisements, and Vendors in a Sample Area of 38.3 km^2^, Soweto, South Africa, 2013

Type of Pattern	Point Type	No. of Points	Intensity (Points/km^2^)
Point	SSB	137	3.58
Covariate	School	28	0.73
	Vendor	184	4.81

Table 3 presents the intensity of the SSB and vendor point patterns against the distance to the nearest school. Intensity of SSB and vendor point patterns increases with proximity to the nearest school. The results of the Kolmogorov–Smirnov test indicated that the SSB (*D* = 0.98, *P* < .001) and vendor (*D* = 0.43, *P* < .001) point patterns were dependent on the distance to the nearest school. Homogenous and inhomogeneous Poisson models were fitted to the SSB advertisements and vendor point patterns. Results indicated an increase in the intensity of SSB advertisements (risk interval [RI] = −2.17, 95% confidence interval [CI] = −2.62 to −1.72) and the vendor (RI = −2.08, 95% CI = −2.48 to −1.82) point patterns with decreasing proximity to the nearest school. Approximately each square kilometer contained 1 primary or high school, 4 SSB advertisements, and 5 vendors, 3 of which sold SSBs; the most frequent advertisements were for 1 beverage company.

## Discussion

Findings from this study indicate that vendors selling both SSBs and advertisements for SSBs are located in close proximity to primary and high schools in Soweto and that this placement is not random. Approximately each square kilometer contained 1 primary or high school, 4 SSB advertisements, and 5 vendors, 3 of which sold SSBs. The most frequent advertisements were for 1 beverage company.

This is the first study of its kind in one of the most densely populated urban areas in South Africa in the process of economic transition. Findings provide an understanding of the obesogenic environment by determining the geospatial intensity and distribution of SSB advertising, as well as the availability of ultra-processed foods and beverages. By strategically positioning advertisements in schoolyards or in close proximity to schools, children are being targeted. In another South African–based study, conducted in Western Cape schools, more than 60% of schools had a branded food or beverage advertisement board displaying the school name (13). Principals of these schools indicated that they had not received any monetary or program support from the sponsoring food and beverage companies, but those advertisements send an implicit message to students and the community (13).

A similar study in Sydney, Australia, found that the most frequent outdoor advertisements in close proximity to schools were for SSBs and alcoholic beverages; 24% of the total number of food advertisements located around these primary schools promoted SSBs (22). In New Zealand, 22% of the outdoor advertisements in close proximity to secondary schools were for SSBs (23). The frequency of these marketing messages influences social norms and promotes the perception that calorie-dense, nutrient-poor beverages and food products are normal (24–26).

To ensure the development of healthy dietary practices, especially in transforming low- and middle-income households in urban areas of South Africa, resources and efforts should be directed toward preventing obesity in these communities and understanding the causes of social determinants of obesity at a population level (27). Policy makers should consider developing mandatory regulations that target advertising in and around schools (28). This type of regulation has a precedent in the example of legislation to restrict tobacco advertising (23). However, an unintended consequence of restrictions placed on outdoor advertising may encourage a switch by industry to other modalities of advertising, such as television or social media (27).

Preventing obesity cannot be solved with a single solution, and managing the obesity epidemic requires efforts at both the population and individual levels (1). The South African Department of Health’s National Strategic Plan for Non-Communicable Diseases 2013–2017 calls for intersectoral and multidisciplinary action (1). Unhealthy product promotion should be limited and substituted with the sponsorship of healthy choices (28). A package of interventions directed toward making the environment healthier and making healthy eating a norm is needed (28). One of these interventions includes a ban on advertising ultra-processed products during television viewing hours for children. Others might involve regulating food and beverages in school vending machines. Finally, tuckshops could ensure that healthy, balanced school lunches are provided (29).

People feel most empowered when they make their own decisions. Children in particular are disempowered and are unable to negotiate the advertising content to which they are exposed (25). Ideally, prohibiting advertising of SSBs to children should be voluntary. If this does not occur in a short period, government should consider setting mandatory standards for the marketing of beverages and food to children and adolescents, as is already occurring in many world settings. The South African government released a set of draft guidelines on the labeling and advertising of food and beverages to children in 2014. Our research supplies data that will provide policy makers with evidence as they move forward (29). Given the growing burden of obesity in South Africa and the challenges of losing weight after adolescence, establishing these standards is now a matter of urgency (30).

A limitation of this study was that it focused on exploring the density and nature of outdoor advertising and did not account for other forms of advertising exposure, such as television, radio, telephone messaging, and print media. Further research is needed to develop a comprehensive picture of the exposure of children to advertising in other formal and informal settings and in rural and urban settings. In addition, the study determined the geospatial intensity and distribution of advertising and availability of SSBs but did not determine a causal relationship between these factors and the prevalence of obesity in Soweto. However, other studies have identified the effect of food availability and advertising on consumption patterns. Our findings have implications for policies that regulate SSB advertising, especially in the proximity of schools.
